# A Clinical Prediction Model for Patients with Acute Large Vessel Occlusion Due to Underlying Intracranial Atherosclerotic Stenosis

**DOI:** 10.1007/s00062-022-01241-3

**Published:** 2022-12-15

**Authors:** Yusen Cai, Yuting Gu, Yanhong Wang, Peng Wang, Lei Zhang, Chaolai Liu, Jianfeng Chu, Hui Li, Zhe Lu, Yafei Zhou, Huakun Liu

**Affiliations:** 1 Jining Medical University, Shandong, China; 2 Women’s & Children’s health care hospital of LinYi, LinYi, China; 3 Jining No,1 People’s Hospital, Shandong, China

**Keywords:** Acute ischemic stroke, Large vessel occlusion, Endovascular treatment, Prediction model, Nomograms

## Abstract

**Background:**

Acute large vessel occlusion due to underlying intracranial atherosclerotic stenosis (ICAS-LVO) increases the difficulty of revascularization, resulting in frequent re-occlusion. The establishment of its pathogenesis before endovascular treatment (EVT) is beneficial for patients. We aimed at developing and validating a clinical prediction model for ICAS-LVO patients before EVT.

**Methods:**

Patients with acute large vessel occlusion at Jining No. 1 People’s Hospital from January 2019 to September 2021 were retrospectively included as the training cohort. The 70 patients who met the inclusion and exclusion criteria were included in the validation cohort (October 2021 to May 2022). Demographics, onset form, medical history, digital subtraction angiography (DSA) imaging data, and laboratory test data were collected. Preprocedural parameters for the ICAS-LVO risk prediction model were established by stepwise logistic regression controlling for the confounding effects. Then, we constructed a nomogram model and evaluated its performance via the Hosmer-Lemeshow goodness-of-fit test, area under the ROC curve (AUC) analysis.

**Results:**

The 231 acute LVO patients were included in the final analysis, 74 (32.3%) patients were ICAS-LVO. A preoperative diagnosis prediction model consisting of five predictors for ICAS-LVO, including fluctuating symptoms, NIHSS < 16, atrial fibrillation, tapered sign, and ASITN/SIR score ≥ 2. The model depicted an acceptable calibration (Hosmer-Lemeshow test, *p* = 0.451) and good discrimination (AUC, 0.941; 95% confidence interval, 0.910–0.971). The optimal cut-off value for the ICAS-LVO scale was 2 points, with 86.5% sensitivity, 91.1% specificity, and 90.5% accuracy. In the validation cohort, the discriminative ability was promising with an AUC value of 0.897, implying a good predictive performance.

**Conclusion:**

The established ICAS-LVO scale, which is composed of five predictors: fluctuating symptoms, NIHSS < 16, atrial fibrillation, tapered sign, and ASITN/SIR score ≥ 2, has a good predictive value for ICAS-LVO in Chinese populations.

## Introduction

Globally, acute ischemic stroke (AIS) due to large vessel occlusion (LVO) is among the leading causes of mortality and disability. Several randomized controlled trials [1–5] have shown that mechanical thrombectomy (MT) can effectively and safely improve the 90-day clinical outcomes for patients with anterior circulation acute ischemic stroke caused by LVO. It has been reported that MT is a potential option for treatment of acute vertebrobasilar artery occlusion [6–8]. Currently, endovascular treatment (EVT) has become the standard treatment for acute ischemic stroke in patients with LVO [9]; however, treatment outcomes of EVT for acute ischemic stroke with LVO are not always ideal. Shorter times to recanalization and higher recanalization rates are important for favorable prognostic outcomes [10].

Large vessel occlusion due to intracranial atherosclerosis (ICAS-LVO) is prevalent among Asians, and the incidences are higher than those found in other areas of the world [11]. Compared to LVO caused by embolism, stent–retriever and aspiration thrombectomy techniques are less efficacious in ICAS-LVO [12], with a markedly low recanalization rate, high re-occlusion rate and longer puncture-to-reperfusion time during endovascular procedures. Successful revascularization requires rescue treatment with intra-arterial thrombolysis, balloon angioplasty, or stenting [13–15]. Establishment of its pathogenesis before interventional surgery will inform on targeted treatment and help to have a smooth procedure.

Unique pathogenesis is associated with more complex and time-consuming surgical procedures, which present significant challenges in ICAS-LVO intervention [16, 17]. Therefore, a new, accurate and comprehensive predictive modality is required for patients with AIS due to ICAS-LVO. In this study, we developed and validated a clinical prediction model for EVT patients.

## Methods

### Patient Selection

We retrospectively evaluated patients who had been subjected to emergency EVT due to acute large vessel ischemic stroke at Jining No. 1 People’s Hospital stroke center from January 2019 to September 2021. The EVT procedures included stent retrieval, aspiration, angioplasty, stenting or a combination of these techniques.

ICAS-LVO was defined as: (1) intracranial artery fixed stenosis of > 70% when successful reperfusion was achieved and (2) degree of intracranial artery stenosis > 50% in addition to either flow and perfusion impairment on angiography or evident re-occlusion tendency even after adequate treatment with stent retrievers. Embolic-LVO was defined as the absence of residual stenosis in occluded segments after successful reperfusion of the occlusion vessel. The exclusion criteria were: (1) the pathogenesis of occluded vessels due to failed recanalization cannot be reliably assessed, (2) when the cause of vascular occlusion was vasculitis, or moyamoya disease, (3) presence of tandem lesions associated with the carotid or vertebral arteries in the extracranial segment (including extracranial stenosis of the vertebral or carotid artery, carotid dissection) and (4) DSA image information is missing. This retrospective, observational study was approved by the Ethical Committee of No. 1 People’s Hospital of Jining.

### Data Collection

Immediately after admission, all patients were subjected to routine blood tests, serum biochemistry tests, blood coagulation tests, electrocardiogram, brain CT NIHSS, mRS, and GCS. Data on demographics, onset form, medical history, digital subtraction angiography (DSA) imaging and laboratory tests were retrospectively collected. Then, data were double entered using the EpiData Entry software v3.1 (EpiData Association, Odense, Denmark). To eliminate selection bias, radiologic assessments were conducted by two neuroradiologists and one neurologist.

### Predictive Factors

Fluctuating symptoms are defined as symptomatic fluctuations and progressive aggravation during the early course of neurologic deficits [18]. The tapered sign denotes occlusive clot signs, described as the appearance of a tapered beak-like or flame-like sign [19, 20]. The American Society of Interventional and Therapeutic Neuroradiology/Society of Interventional Radiology (ASITN/SIR) developed the collateral flow grading scale for DSA [21] to evaluate collateral circulation. The National Institute of Health stroke scale (NIHSS) is a comprehensive stroke scale for evaluating neurological deficits.

### Statistical Analyses

Multiple sixfold imputation was performed using the chained equations method to fill the missing blood test results (missing values < 5%). Baseline characteristics were compared between the ICAS-LVO and embolic LVO groups. Categorical variables are presented as numbers and percentages *N* (%) and compared using the χ^2^-testand Fisherʼs^,^ exact test. Continuous variables are presented as mean ± standard deviation if normally distributed and as medians and interquartile ranges (IQR), if not normally distributed. The Mann-Whitney U test and the t‑test were used to compare continuous variables between groups.

Multivariable logistic regression analysis was performed for variables with *p* < 0.05 in the univariate regression analysis. Cut-off values were determined using the maximum of Youden index. Continuous variables were translated into categorical variables. Collinearity diagnosis was performed using the variance inflation factor (VIF). Binary logistic regression (forward, backward) was used to evaluate the predictors and to generate the regression model as well as its related odds ratio (OR), 95% confidence interval (CI), beta coefficient and *p* values. The beta coefficients were rounded to the closest integer to generate a brief ICAS-LVO scale. The ROC curve was used to calculate the optimal cut-off value of the area under the curve (AUC) to assess the predictive accuracy of the scale and discriminatory ability of the model. The Hosmer-Lemeshow test was performed to assess calibration. A nomogram was built on the brief predictive model as a graphical presentation.

#### Predictive Models

Analyses were performed using the SPSS software package, version 22 (IBM, Armonk, NY, USA) and R software, version 4.1.3 (R Statistical Software, R Foundation for Statistical Computing, Vienna, Austria).

**Fig. 1:**
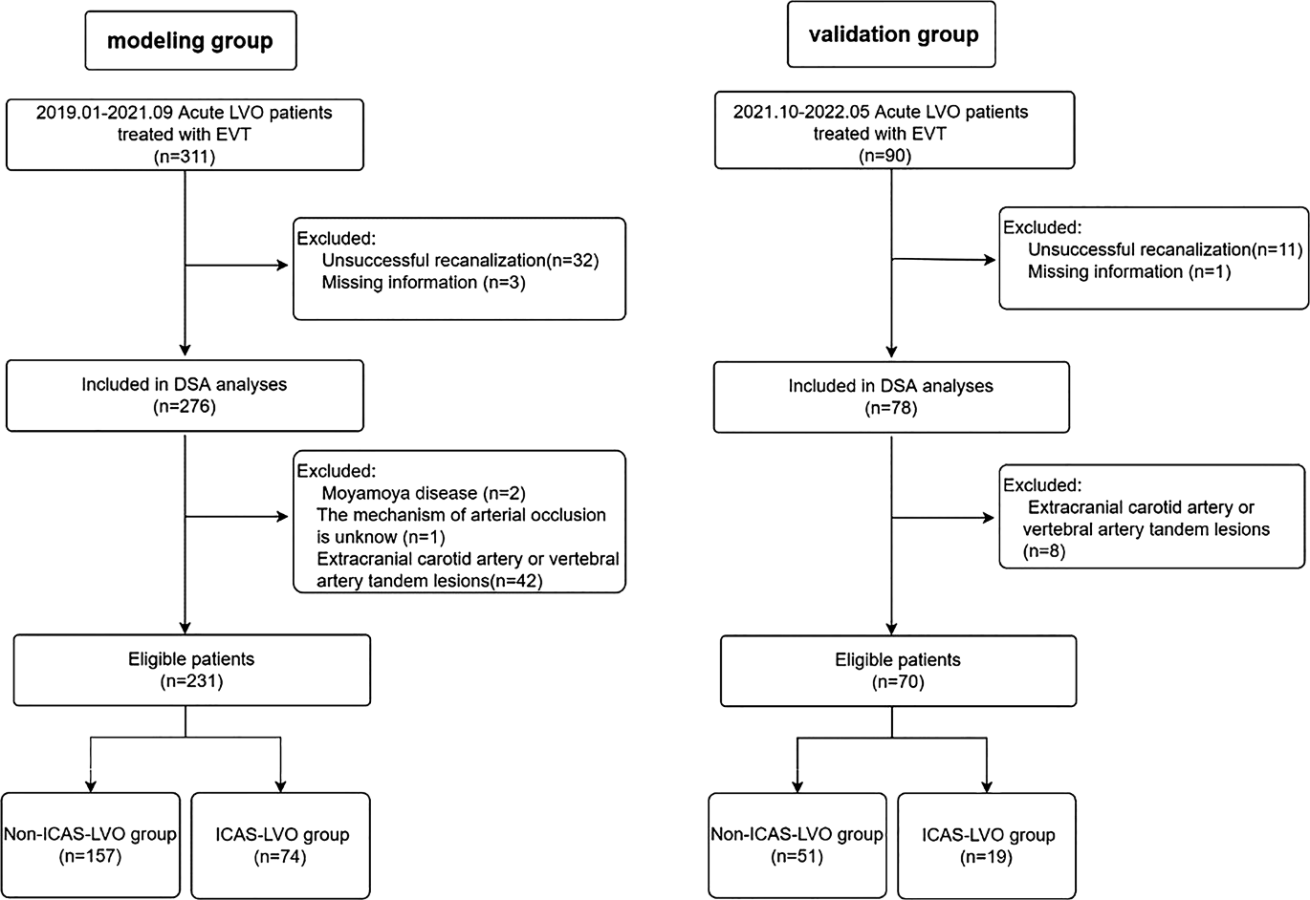
Experimental flow chart. *LVO* Large vessel occlusion, *EVT* endovascular treatment, *DSA* digital subtraction angiography, *ICAS* Intracranial atherosclerosis

## Results

**Fig. 2:**
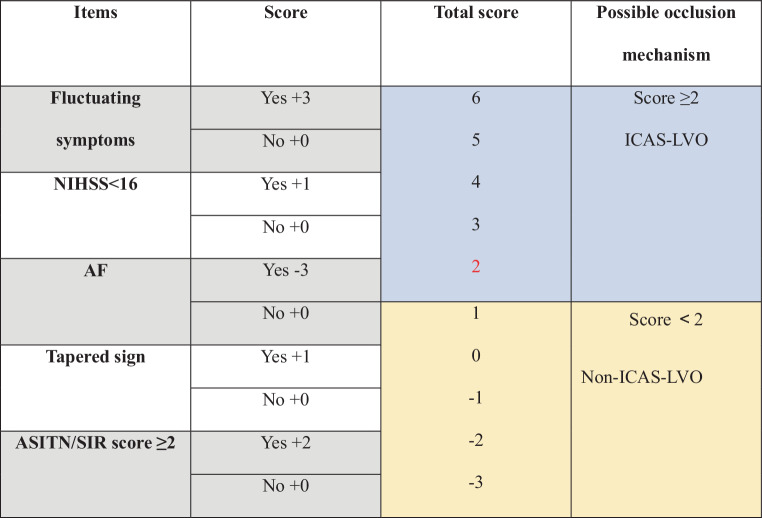
Schematic presentation of the ICAS-LVO prediction model. *NIHSS* National Institutes of Health stroke scale, *AF* atrial fibrillation, *ASITN/SIR score* American Society of Interventional and Therapeutic Neuroradiology/Society of Interventional Radiology, *β‑coefficient* the regression coefficient, *OR* odds ratios, *CI* confidence interval

Tables 1, 2 and 4; Figs. 2 and 4.

**Table 1:** Baseline characteristics of the modelling group

Variables	ICAS-LVO group (*n* = 74)	Embolic group (*n* = 157)	*p*-values
Age, (years, mean (SD))	63.46 ± 9.24	65.78 ± 12.44	0.140
Gender, (male, *n* (%))	57 (77.00%)	106 (67.50%)	0.284
SBP, (mm Hg, median [IQR])	157.53 ± 23.78	149.41 ± 23.44	0.016
DBP, (mm Hg, median [IQR])	93.50 [80.00,102.00]	85.00 [75.00, 95.00]	0.001
**Medical history**			
Hypertension, (*n* (%))	13 (68.42%)	23 (45.10%)	0.083
Diabetes, (*n* (%))	30 (40.50%)	35 (22.30%)	0.009
CHD, (*n* (%))	3 (15.79%)	11 (32.50%)	0.840
AF, (*n* (%))	4 (5.40%)	83 (52.90%)	< 0.001
VHD, (*n* (%))	1 (1.40%)	18 (11.50%)	0.090
ICAS, (*n* (%))	8 (10.80%)	6 (3.80%)	0.075
Ischemic stroke, (*n* (%))	15 (20.30%)	35 (22.30%)	0.728
ICH, (*n* (%))	1 (1.40%)	5 (3.20%)	0.708
Smoking, (*n* (%))	30 (40.50%)	42 (26.90%)	0.037
Drinking, (*n* (%))	30 (40.50%)	42 (26.80%)	0.035
**Onset form**			
Fluctuating symptoms, (*n* (%))	46 (62.20%)	9 (5.7%)	< 0.001
**Scores after admission**			
NIHSS score, (median [IQR])	15 [12.00, 20.00]	19 [16.00, 21.50]	0.001
GCS score, (median [IQR])	10 [8.00, 12.00]	9 [6.00, 11.00]	0.120
MRS score, (median [IQR])	4 [4.00, 5.00]	5 [4.00, 5.00]	0.423
ASPECT (pr-ASPECT), (median [IQR])	7.37 [7.00, 8.00]	7.37 [7.00, 8.00]	0.305
ASITN/SIR score (median [IQR])	2.00 [1.00, 3.00]	1.00 [0.00, 1.00]	< 0.001
**Occlusive clot signs**			
Cut-off sign	18 (24.30%)	70 (46.40%)	0.010
Claw sign	11 (14.90%)	37 (24.70%)	0.093
Meniscus sign	2 (2.70%)	15 (8.70%)	0.163
Tram-track sign	22 (29.70%)	16 (10.70%)	0.001
Tapered sign	19 (25.70%)	14 (14.70%)	0.010

*SD* standard deviation, *SBP* systolic blood pressure, *IQR* interquartile range, *DBP* diastolic blood pressure, *CHD* coronary heart disease, *AF* atrial fibrillation, *VHD* heart valve disease, *ICAS* intracranial artery stenosis, *ICH* intracerebral hemorrhage, *NIHSS* National Institutes of Health stroke scale, *GCS* Glasgow Coma Scale, *MRS* modified Rankin scale, *ASPECTS* Acute Stroke Programme Early Computed Tomography Score, *pc-ASPECTS* posterior circulation Acute Stroke Programme Early Computed Tomography Score, *ASITN/SIR score* ASITN/SIR American Society of Interventional and Therapeutic Neuroradiology/Society of Interventional Radiology

**Table 2:** Laboratory findings of the modelling group

Variables	ICAS-LVO Group (*n* = 74)	Embolic-LVO Group (*n* = 157)	*p*-value
WBC (10^9^ × /L) median [IQR]	11.19 [8.29, 13.57]	9.53 [7.02, 12.51]	0.035
Hb (g/L) median [IQR]	139.00 [126.75, 148.00]	132.00 [119.00, 147.68]	0.106
Platelets (10^9^ × /L) mean ± (SD)	245.67 ± 60.60	207.72 ± 64.56	0.540
HbA1 mean ± (SD)	6.89 ± 1.74	6.34 ± 1.68	0.510
Neutrophils (×10^9^/L) median [IQR]	9.80 [6.45, 11.1]	7.49 [5.56, 11.40]	0.040
Lymphocytes (×10^9^/L) median [IQR]	1.07 [0.80, 1.58]	1.26 [0.81, 1.93]	0.194
NLR median [IQR]	8.47 [5.07, 11.83]	6.40 [3.80, 10.96]	0.037
VLDL (mmol/L) median [IQR]	0.71 [0.43, 1.10]	0.52 [0.31, 0.73]	0.002
LDL (mmol/L) mean ± (SD)	2.79 ± 1.14	2.33 ± 0.85	0.010
TC (mmol/L) mean ± (SD)	4.72 ± 1.20	4.07 ± 1.08	0.090
TG (mmol/L) median [IQR]	1.60 [1.03, 2.41]	1.12 [0.73, 1.63]	0.001
HDL (mmol/L) median [IQR]	1.03 [0.90, 1.38]	1.09 [0.95, 1.31]	0.896
Apo A (nmol/L) median [IQR]	27.85 [13.20, 68.60]	25.90 [11.20, 67.37]	0.379
SOD (U/mL) median [IQR]	138.50 [124.00, 157.00]	137.00 [120.00, 152.00]	0.581
Fibrinogen (g/L) median [IQR]	2.60 [2.06, 3.43]	2.53 [2.08, 3.12]	0.316
INR median [IQR]	1.01 [0.94, 1.08]	1.07 [1.01, 1.15]	0.012
APTT(s) median [IQR]	25.05 [23.00, 29.38]	26.10 [23.00, 31.50]	0.451
PT median [IQR]	11.55 [10.90, 12.40]	12.40 [11.25, 14.20]	< 0.001
TT (s) median [IQR]	18.30 [16.38, 21.23]	17.60 [16.10, 22.40]	0.499
Procalcitonin median [IQR]	0.05 [0.05, 0.12]	0.05 [0.05, 0.11]	0.337

*WBC* white blood cells, Hb hemoglobin, *HbA1c* glycated hemoglobin, *NLR* NLR neutrophil-to-lymphocyte ratio, *VLDL* very low-density lipoproteins, *LDL* low-density lipoproteins, *HDL* high-density lipoproteins, *TG* triglyceride, *TC* total cholesterol, *Apo A* lipoprotein A, SOD superoxide dismutase, *PT* prothrombin time, *TT* thrombin time, *APTT* activated partial thromboplastin time, *INR* international normalized ratio

**Table 3:** Predictive factors for ICAS-LVO

Variable	β‑coefficient	Standard error	Wald	OR (95%CI)	*p*-value
Fluctuating symptoms	3.071	0.599	26.245	21.56 (6.66, 69.8)	< 0.001
NIHSS < 16	1.287	0.479	7.220	3.62 (1.42, 9.3)	0.007
AF	−3.312	0.717	21.321	0.04 (0.01, 0.1)	< 0.001
ASITN/SIR score	2.362	0.504	22.005	10.61 (3.96, 28.5)	< 0.001
Tapered sign	1.491	0.677	4.850	4.44 (1.18, 16.8)	0.028

*NIHSS* National Institute of Health stroke scale, *AF* atrial fibrillation, *ASITN/SIR score* ASITN/SIR American Society of Interventional and Therapeutic Neuroradiology/Society of Interventional Radiology, *β‑coefficient* the regression coefficient, *OR* odds ratio, *CI* confidence interval

**Table 4:** Baseline characteristics of the validation group

Variables	ICAS-LVO group (*n* = 19)	Embolic-LVO group (*n* = 51)	*p*-value
Age, (year, mean (SD))	62.68 ± 8.62	63.24 ± 12.55	0.861
Male, (*n* (%))	9 (47.37%)	35 (68.63%)	0.102
SBP, (mm Hg, median [IQR])	151.11 ± 23.54	146.76 ± 25.72	0.523
DBP, (mm Hg, median [IQR])	93.50 [80.00, 93.00]	86.58 [80.00, 98.00]	0.592
**Medical history**			
Hypertension, (*n* (%))	13 (68.42%)	23 (45.10%)	0.083
Diabetes, (*n* (%))	4 (21.05%)	10 (19.6%)	0.980
CHD, (*n* (%))	3 (15.79%)	11 (21.57%)	0.840
AF, (*n* (%))	1 (5.26%)	26 (50.98%)	< 0.001
VHD, (*n* (%))	0 (0.00%)	4 (7.84%)	0.498
ICAS, (*n* (%))	1 (5.26%)	1 (1.96%)	0.472
Ischemic stroke, (*n* (%))	3 (15.79%)	6 (11.76%)	0.963
ICH, (*n* (%))	0 (0.00%)	2 (3.92%)	0.528
Smoking, (*n* (%))	8 (42.10%)	13 (25.49%)	0.177
Drinking, (*n* (%))	4 (21.05%)	13 (25.49%)	0.943
**Onset form**			
Fluctuating symptoms, (*n* (%))	46 (62.20%)	9 (5.7%)	< 0.001
**Scores after admission**			
NIHSS score < 16, (*n* (%))	13 (68.42%)	15 (29.41%)	0.003
GCS score, (median [IQR])	10 [8.00, 12.00]	9 [6.00, 11.00]	0.120
MRS score, (median [IQR])	4 [4.00, 5.00]	5 [4.00, 5.00]	0.423
ASITN/SIR score ≥ 2, (*n* (%))	14 (73.68%)	13 (25.49)	0.010
**Occlusive clot signs**			
Tapered sign	16 (25.70%)	5 (9.80%)	0.020

*SD* standard deviation, *SBP* systolic blood pressure, *IQR* interquartile range, *DBP* diastolic blood pressure, *CHD* coronary heart disease, *AF* atrial fibrillation, *VHD* heart valve disease, *ICAS* intracranial artery stenosis, *ICH* intracerebral hemorrhage, *NIHSS* National Institute of Health stroke scale, *GCS* Glasgow Coma Scale, *MRS* modified Rankin Scale, *ASITN/SIR score* ASITN/SIR American Society of Interventional and Therapeutic Neuroradiology/Society of Interventional Radiology

**Fig. 3:**
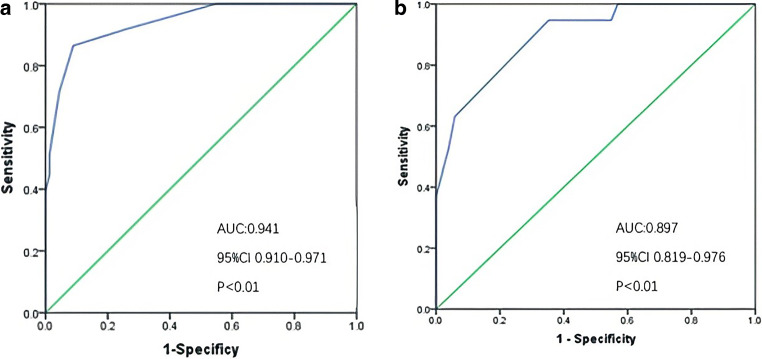
The ROC curves of the ICAS-LVO predictive scale. **a** The ROC curves for modelling group, AUC: 0.941, 95% CI 0.910–0.971, *p* < 0.01; **b** The ROC curves for validation group, AUC: 0.897, 95% CI 0.819–0.976, *p* < 0.01. *AUC* area under curve, *CI* confidence interval

**Fig. 4:**
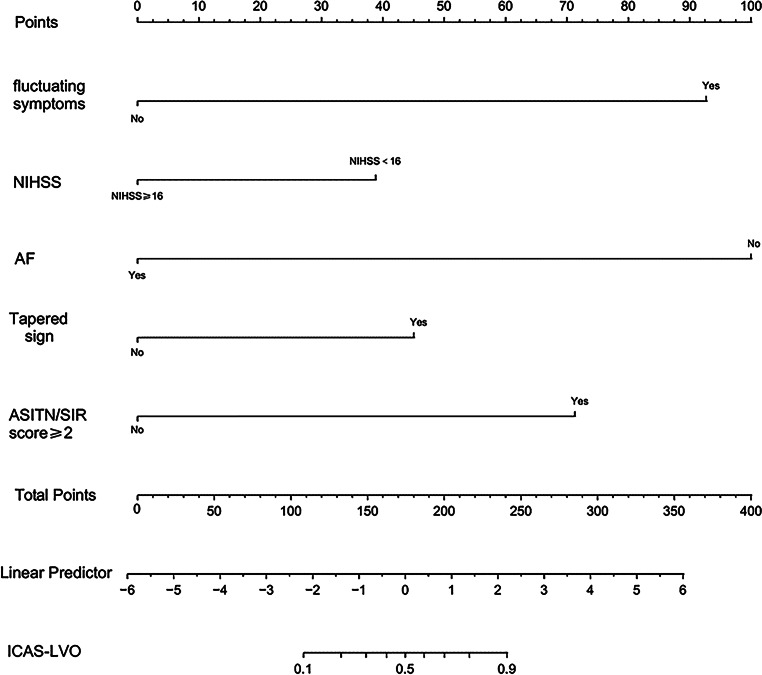
Nomogram for predicting the probability of ICAS-LVO in patients with acute ischemic stroke. *NIHSS* National Institutes of Health stroke scale, *AF* atrial fibrillation, *ASITN/SIR score* American Society of Interventional and Therapeutic Neuroradiology/Society of Interventional Radiology, *ICAS-LVO* acute large vessel occlusion due to intracranial atherosclerosis

### Patient Baseline Characteristics

From January 2019 to September 2021, a total of 311 consecutive AIS patients with LVO were enrolled in the modelling group. Of the patients 32 were excluded because the pathogenesis of occluded vessels could not be reliably assessed due to unsuccessful revascularization, while 3 were excluded because of missing DSA image information. Then, 272 patients were subjected to DSA analyses. Based on vascular occlusive mechanisms, 45 patients (moyamaya disease *n* = 2, tandem lesions associated with the carotid or vertebral arteries in the extracranial segment *n* = 42, including extracranial stenosis of the vertebral or carotid artery carotid dissection *n* = 35, extracranial carotid dissection *n* = 7) were excluded. Finally, 231 patients were analyzed (Fig. 1).

Baseline characteristics for the training cohort are presented in Table 1. The median age for all participants was 65 ± 11.54 years and the number of males was 163 (70.56%); baseline NIHSS 18.0 (IQR 15.0, 21.0); GCS score 9.0 (IQR 7, 12); mRS score on admission 5.0 (IQR 4.0, 5.0); ASPECTS (pr-ASPECTS) 7 (IQR 7.0, 8.0); ASITN/SIR score 1.0 (IQR 0.0, 2.0). Risk factors included history of hypertension (*n* = 122, 52.81%); diabetes (*n* = 65, 28.14%); CHD *n* = 67 (29.00%); AF *n* = 87 (37.66%); VHD *n* = 19 (8.25%); ICAS *n* = 14 (6.06%); ischemic stroke *n* = 50 (21.65%); ICH, *n* = 6 (2.60%); smoking *n* = 72 (31.17%); and drinking 72 (*n* = 31.17%). Based on findings from DSA, patients were divided into two groups: ICAS-LVO group 74 (32.04%) and embolic-LVO group 157 (67.96%).

The ICAS-LVO and embolic-LVO groups differed in various aspects (Tables 1 and 2). Compared to the embolic-LVO group, patients with ICAS-LVO were younger, male, had higher blood pressure and blood lipid levels at admission (SBP:157.53 mmHg ± 23.78 mmHg vs. 149.41 mmHg ± 23.44 mmHg, *p* = 0.016, DBP: 93.50 mmHg [IQR 80.00 mmHg, 102.00 mmHg] vs. 85.00 mmHg [IQR 75.00 mmHg, 95.00 mmHg] *p* = 0.001; VLDL, very-low-density lipoprotein (0.71 mmol/L [IQR 0.43 mmol/L, 1.10 mmol/L] vs. 0.52 mmol/L [IQR 0.31 mmol/L, 0.73 mmol/L], *p* = 0.002; LDL, low density lipoproteins (2.79 mmol/L ± 1.14 mmol/L vs. 2.33 mmol/L ± 0.85 mmol/L, *p* = 0.010); TG, triglyceride (1.60 mmol/L [IQR 1.03 mmol/L, 2.41 mmol/L] vs. 1.12 mmol/L [IQR 0.73 mmol/L, 1.63 mmol/L]), *p* = 0.001) current smoking and drinking (40.50% vs. 26.90%), *p* = 0.037; 40.50% vs. 26.90%, *p* = 0.035), and had lower baseline NIHSS scores 15[IQR 12.00, 20.00] vs. 19[IQR 16.00, 21.50] *p* = 0.001; better collateral circulation ASITN/SIR score 2.00[IQR 1.00, 3.00] vs. 1.00[IQR 0.00, 1.00], *p* < 0.001. The embolic-LVO group had more cases of atrial fibrillation (52.90% vs. 5.40%, *p* < 0.001) and heart valve disease (11.5% vs. 1.40%, *p* = 0.09); INR (1.07[IQR 1.01, 1.15] vs. 1.01 [IQR 0.94, 1.08] *p* < 0.001); PT, (12.40 [IQR 11.25, 14.20] vs. 11.55 [IQR 10.90, 12.40], *p* = 0.001); WBC, (9.53×10^9^/L [IQR 7.02×10^9^/L, 12.51×10^9^/L] vs. 11.19×10^9^/L [IQR 8.29×10^9^/L, 13.57×10^9^/L], *p* = 0.035); neutrophils, (7.49×10^9^/L [IQR 5.56×10^9^/L, 11.40×10^9^/L] vs. 9.80×10^9^/L[IQR 6.45×10^9^/L, 11.1×10^9^/L], *p* = 0.040); NLR (6.40 [IQR 3.80, 10.96] vs. 8.47 [IQR 5.07, 11.83], *p* = 0.037) and a lower proportion of ICAS history (3.80% vs. 10.80%, *p* = 0.003) when compared to the ICAS-LVO group.

The internal carotid artery siphon, and the trunks before bifurcation in the M1 segment of the middle cerebral artery (MCA), mid-lower segment of the basilar artery are the most common intracranial sites of stenosis [22]. In this study, there was a predominance of anterior circulation LVO (163, 70.6%) and ICAS (42, 25.74%). Of the 68 (29.4%) posterior circulation stroke patients, 32 (47.06%) had intracranial stenosis. Specific sites for stenosis among the 74 ICAS patients were: M1 segments of the MCA (34, 45.95%), communicating segment of the internal carotid artery (3, 4.04%), ophthalmic segment of internal carotid artery (5, 6.76%), middle and lower segments of basilar artery (19, 25.68%) and V4 segment of the vertebral artery (13, 17.57%).

After excluding factors with collinearity or clinical relations with others, there were five more variables in the prediction model: fluctuating symptoms, NIHSS < 16 (cut-off value, 16), AF, tapered, ASITN/SIR score ≥ 2 (cut-off value, 2). The model depicted acceptable calibration (Hosmer-Lemeshow test, *p* = 0.451) and good discrimination (AUC, 0.941; 95% CI, 0.910–0.971). The β‑coefficients of the five predictors are shown in Table 3. The optimal cut-off value for the ICAS-LVO scale was 2 points with 86.5% sensitivity, 91.1% specificity, and 90.5% accuracy (Fig. 2). ICAS-LVO scale was developed as a nomogram (Fig. 4).

### Validation Group

A total of 70 patients were included in the final analysis, ICAS-LVO (19, 27.14%) the discrimination ability was still promising with an AUC value of 0.897 and a good predictive performance (Fig. 3; Table 4).

## Discussion

Early and accurate diagnosis of ICAS-LVO before interventional surgery is particularly important to help select the most appropriate device [23]. It is well known that preoperative etiology is relatively difficult to predict. In particular, vascular occlusion of intracranial segments. Tandem occlusion due to atherosclerotic stenosis of extracranial arteries or a carotid dissection is easily recognized early based on anatomical structures and unique imaging [24–26]. Currently, some predictors have been shown to distinguish between ICAS-LVO and embolism LVO with acute ischemic stroke patients. The high-resolution vessel wall magnetic resonance imaging (MRI) has limited applications in assessment of LVO type due to delays in treatment [27–29]. Some of the predictive studies were based on intraprocedural angiographic signs (IPASs) [19]. Although microcatheter “first-pass effects” exhibit reliable outcomes in identification of ICAS, they need a microcatheter through the area of total occlusion, which seems lacking in predicting models [30].

Tapered sign is defined as the appearance of a tapered beak-like or flame-like sign on DSA imaging [31–33]. Tapered signs are vital for identifying ICAS before an operation, but also present in occlusions due to arterial dissection [34]. Tapered signs in the petrocavernous segment of ICA or the origin of basilar artery are poorly predictive of ICAS-LVO [20]. ICAS-LVO patients exhibit a unique clinical history, including frequently present progressive or fluctuating symptoms [18], better collateral circulation [23], hypertension, diabetes, smoking [22], lower admission NIHSS score [35] and are younger than embolic-LVO group. A pre-EVT in situ atherosclerotic thrombosis (ISAT) predictive model formulated by Xing Jin et al. consists of three predictive factors: history of hypertension, atrial fibrillation rhythm, and dichotomous serum glucose levels. The ISAT scale is only applicable to patients with acute vertebrobasilar arterial occlusion [36]. Compared with previous studies, our study focused on the preoperative judgment of ICAS-LVO. The higher predictive value of the model helps in the accurate identification of ICAS in intracranial occluded segments, ease to implement and promotion.

Collateral circulation plays an important role in preserving perfusion and stabilizing cerebral blood flow in acute occlusion [37]. Chronic atherosclerotic intracranial arterial stenosis may lead to a compensatory adjustment in the brain. Collateral circulation has been shown to be better in patients with ICAS-LVO than in those without chronic stenosis, implying that good collateral circulation is a predictor of ICAS-LVO [37–39]. There is a significant association between angiographic collateral scores and baseline NIHSS scores [40].

We included variables that were associated with ICAS-LVO as predictors into the analysis. Binary logistic stepwise regression analysis was performed to evaluate the predictors and generate the ICAS-LVO scale, which had 5 predictors; fluctuating symptoms, NIHSS < 16, AF, tapered sign, and ASITN/SIR score ≥ 2. These 5 predictors are easily available clinically. This scale was shown to have the ability to identify the pathogenesis of intracranial vascular occlusion before interventional therapy and to enhance the identification of acute cerebral infarction due to ICAS-LVO. It is important for neurointerventionalists to perform optimal revascularization strategies to reduce the difficulty of surgery and shorten the vascular recanalization time, although, the preferred initial treatment during interventional therapy for ICAS-LVO patients has yet to be established; however, the identification of ICAS-LVO before EVT may improve recanalization rates with more targeted revascularization techniques and shorten recanalization times with early remedial measures [20, 36, 41]. The primary goal of recanalization is to rapidly open the occluded artery and remove the clot. The structure of intracranial atherosclerosis occlusive lesions is different from cardiogenic emboli [23]. Aspiration catheter recanalization for recanalization of ICAS-LVO exhibited poor outcomes [42, 43]. Stent retrievers as a first-line device may achieve a higher success rate than suction catheters in patients with ICAS-LVO [41]; however, repeated stent retrievers thrombectomy is associated with the possibility of intima injuries around the ICAS, which activates the platelets, easily resulting in occlusion again [44]. To eliminate potential stenosis and prevent re-occlusion, nearly half of ICAS-LVO patients require angioplasty with or without stent implement rescue treatment. Rescue treatment is a complex and time-consuming process that requires individualized treatment strategies [45–47].

## Limitations

There are several limitations in this study. First, as a retrospective observational study conducted in a single center, there may be information bias. Second, the clinical prediction models drawn from this study are limited by the relatively small sample size. Our findings should be verified via further external validation and their generalizability evaluated using large sample, multicenter datasets.

## Conclusion

The predictive scale comprising of fluctuating symptoms, NIHSS < 16, atrial fibrillation, tapered sign, and ASITN/SIR score ≥ 2 has a promising predictive value for ICAS-LVO before EVT in ischemic stroke due to acute large-vessel occlusion patients.
